# Independent External Validation of FRAX and Garvan Fracture Risk Calculators: A Sub‐Study of the FRISBEE Cohort

**DOI:** 10.1002/jbm4.10532

**Published:** 2021-08-06

**Authors:** Felicia Baleanu, Laura Iconaru, Alexia Charles, Virginie Kinnard, Jean‐François Fils, Michel Moreau, Rafik Karmali, Murielle Surquin, Florence Benoit, Aude Mugisha, Marianne Paesmans, Michaël R Laurent, Pierre Bergmann, Jean‐Jacques Body

**Affiliations:** ^1^ Department of Endocrinology, CHU Brugmann Université Libre de Bruxelles Brussels Belgium; ^2^ Department of Clinical Research, CHU Brugmann Université Libre de Bruxelles Brussels Belgium; ^3^ Department of Geriatrics, CHU Brugmann Université Libre de Bruxelles Brussels Belgium; ^4^ Ars Statistica Nivelles Belgium; ^5^ Data Centre, Institute Jules Bordet Université Libre de Bruxelles Brussels Belgium; ^6^ Centre for Metabolic Bone Diseases University Hospitals Leuven Leuven Belgium; ^7^ Imelda Hospital Bonheiden Belgium; ^8^ Department of Nuclear Medicine, CHU Brugmann Université Libre de Bruxelles Brussels Belgium

**Keywords:** BMD, DISCRIMINATION, FRACTURE, OSTEOPOROSIS, RISK ASSESSMENT, RISK FACTORS, VALIDATION

## Abstract

Probabilistic models including clinical risk factors with or without bone mineral density (BMD) have been developed to estimate the 5‐ or 10‐year absolute fracture risk. We investigated the performance of the FRAX and Garvan tools in a well‐characterized population‐based cohort of 3560 postmenopausal, volunteer women, aged 60 to 85 years at baseline, included in the Fracture Risk Brussels Epidemiological Enquiry (FRISBEE) cohort, during 5 years of follow‐up. Baseline data were used to calculate the estimated 10‐year risk of hip and major osteoporotic fractures (MOFs) for each participant using FRAX (Belgium). We computed the 5‐year risk according to the Garvan model with BMD. For calibration, the predicted risk of fracture was compared with fracture incidence across a large range of estimated fracture risks. The accuracy of the calculators to predict fractures was assessed using the area under the receiver operating characteristic curves (AUC). The FRAX tool was well calibrated for hip fractures (slope 1.09, *p* < 0.001; intercept −0.001, *p* = 0.46), but it consistently underestimated the incidence of major osteoporotic fractures (MOFs) (slope 2.12, *p* < 0.001; intercept −0.02, *p* = 0.06). The Garvan tool was well calibrated for “any Garvan” fractures (slope 1.05, *p* < 0.001; intercept 0.01, *p* = 0.37) but largely overestimated the observed hip fracture rate (slope 0.32, *p* < 0.001; intercept 0.006, *p* = 0.05). The predictive value for hip fractures was better for FRAX (AUC: 0.841, 95% confidence interval [CI] 0.795–0.887) than for Garvan (AUC: 0.769, 95% CI 0.702–0.836, *p* = 0.01). The Garvan AUC for “any Garvan” fractures was 0.721 (95% CI 0.693–0.749) and FRAX AUC for MOFs was 0.708 (95% CI 0.675–0.741). In conclusion, in our Belgian cohort, FRAX estimated quite well hip fractures but underestimated MOFs, while Garvan overestimated hip fracture risk but showed a good estimation of “any Garvan” fractures. Both models had a good discriminatory value for hip fractures but only a moderate discriminatory ability for MOFs or “any Garvan” fractures. © 2021 The Authors. *JBMR Plus* published by Wiley Periodicals LLC on behalf of American Society for Bone and Mineral Research.

## Introduction

About one of three women and one of five men worldwide suffer an osteoporotic fracture after the age of 50 years, with major consequences on morbidity, mortality, and health care costs.^(^ ^1^, ^2^ ^)^ Because efficient treatments are available to decrease this fracture risk, people at high fracture risk who are candidates for such treatments should be identified. Bone mineral density (BMD) measured by dual‐energy X‐ray absorptiometry (DXA) at the spine or hip is strongly associated with the incidence of osteoporotic fractures, with a risk gradient of 2.0 to 2.5 per standard deviation (SD) decrease. However, the positive predictive value of BMD is low, since about only half of fragility fractures occur in subjects with osteoporosis, according to the operational WHO definition of osteoporosis (*T*‐score ≤ −2.5).^(^ ^3^ ^)^ Thus, probabilistic models including other clinical risk factors (CRFs) have been developed to estimate the 5‐ or 10‐year absolute fracture risk.

The Garvan fracture risk calculator was developed in 2007 and is based on five risk factors (age, sex, BMD [or body weight], number of prior fractures [0, 1, 2, 3 or more] after 50 years of age, and frequency of falls [0, 1, 2, 3 or more] in the last 12 months) identified from the Dubbo Osteoporosis Epidemiology Study data in 1358 women and 858 men aged 60 years or more. The model can be used with or without BMD to predict 5‐ and 10‐year absolute risk of hip fracture or any fragility fracture (including those of the hip, vertebra [symptomatic], wrist, metacarpal, humerus, scapula, clavicle, distal femur, tibia/fibula, patella, pelvis, ribs, sternum, hands and feet excluding digits—referred further in the text as “any Garvan” fractures).^(^ ^4^ ^)^

In 2008, Kanis and colleagues introduced a country‐specific Fracture Risk Assessment Tool (FRAX), estimating the 10‐year probability of hip fracture or major osteoporotic fractures (MOFs: hip, clinical spine, humerus, and wrist).^(^ ^5^ ^)^ The FRAX model is based upon data collected from nine large international cohorts under contract with the World Health Organization Task Force. In addition to BMD, age, and a history of fragility fracture after age 50 years as in the Dubbo data set, other risk factors demonstrated to be independent predictors of fractures (glucocorticoid use for at least 3 months, low body mass index [BMI], parental history of hip fracture, cigarette smoking, and excessive alcohol intake) were added to calculate fracture risk, taking into account the competitive risk of death. Both the Garvan and FRAX model are available online.

Another prediction tool, Qfracture, which is also available online, was developed based on data from electronic health records from a UK prospective open cohort study. This tool takes into account 32 CRFs among which history of falls and fractures, age and BMI, and different comorbidities and pharmacological treatments, but it does not include BMD.^(^ ^6^ ^)^

Risk prediction tools are increasingly used to estimate individuals' risks of disease in the clinical setting, hence the need to validate them. Validation in populations differing from those used to develop the model, for example by nationality, ethnicity, or specific comorbidities, is necessary to determine their generalizability. The two primary measures used to validate a risk prediction tool are calibration and discrimination. Calibration is the ability to accurately predict the absolute risk level, and discrimination is the ability to accurately rank individuals according to risk.^(^ ^7^ ^)^

FRAX has been validated (by both calibration and discrimination) by 26 studies conducted in nine countries, Garvan by six studies in three countries, and QFracture by three studies within the United Kingdom and the Republic of Ireland only.^(^ ^8^ ^)^

A recent systematic review performed by Beaudoin and colleagues identified 53 validation studies of 14 tools. These studies assessed only the discrimination power. Among these tools, nine were originally developed to predict fracture risk, four were developed for identification of patients with low BMD, one estimated risk of death, and one was conceived to assess frailty. Given the small number of studies on some tools, only FRAX, Garvan, and QFracture were finally compared.^(^ ^9^ ^)^

Because it was most extensively calibrated and validated, the FRAX score is currently the most widely used tool to identify patients at risk for osteoporotic fractures. The Garvan tool is less commonly used, whereas QFracture is used in UK only. A Belgian version of the FRAX based on the national incidence of hip fractures has been published^(^ ^10^ ^)^ but has not been validated yet.

Our primary objective was to evaluate and compare the discrimination power and calibration of the FRAX (Belgium) and Garvan fracture risk prediction tools in a well‐characterized population‐based cohort of 3560 postmenopausal, volunteer women, aged 60 to 85 years at baseline, included in the Fracture Risk Brussels Epidemiological Enquiry (FRISBEE) cohort, during 5 years of follow‐up.

We compared the fracture risk estimated by these calculators with the observed incidence of fractures within 5 years of inclusion and we evaluated the sensitivity and specificity of both models to predict the occurrence of fractures during this period.

Furthermore, we applied to our cohort the National Osteoporosis Foundation (NOF)^(^ ^11^ ^)^ and the Belgian proposed threshold,^(^ ^12^ ^)^ at which treatment intervention becomes cost‐effective, required for appropriate identification of individuals who need treatment and to optimize efficient osteoporosis care.

## Materials and Methods

### Participants and FRISBEE study design

The FRISBEE study, conducted in Brussels (Belgium), is an ongoing prospective population‐based cohort study, aimed at validating and integrating several independent CRFs in order to develop a fracture risk model in a well‐characterized patient population.^(^ ^13^, ^14^ ^)^ Briefly, 3560 postmenopausal, volunteer women, aged 60 to 85 years at baseline, were enrolled between July 2007 and June 2013. Participants were randomly selected from population lists of six districts of Brussels and recruited by postal letter. Baseline characteristics were collected by trained nurses during a face‐to‐face interview. DXA was performed on the same day. Follow‐up data collections are done by annual phone calls and are planned to continue for at least 10 years.

Informed consent was obtained from each woman by return mail. The protocol was accepted by the Ethics Committee of all participating sites (approval number B07720072493).

### Baseline characteristics and fracture ascertainment

Information regarding sociodemographic and anthropometric data, medical history, and current or past medications (including the use of calcium and vitamin D supplements) were registered. We assessed the presence of well‐known CRFs included in the FRAX model (age, body mass index [BMI], prior fragility fracture, parental history of hip fracture, ever use of oral glucocorticoids during a cumulative period of 3 months or longer, rheumatoid arthritis, current smoking, excessive alcohol intake, and any cause of secondary osteoporosis) and other validated CRFs not included in the FRAX model (early non‐substituted menopause [occurring before 45 years] which was recorded as a CRF per se, fall history, activity level, presence of comorbidities, use of selective serotonin reuptake inhibitors [SSRIs] or proton pump inhibitors [PPIs]).

Fall history (typically defined as a fall from standing height or less) was documented using a frequency indicator (no falls, less than one fall per month, less than one fall per week, and more than one fall per week) and time to the last fall (during the previous month, after more than 1 month but within the previous 6 months, after more than 6 months).^(^ ^13^ ^)^

BMD at the lumbar spine level (L_1_ to L_4_) and at the hip (femoral neck, trochanter, and total hip) was measured by DXA (Hologic System 4500 W, Hologic, Inc., Marlborough, MA, USA).

Incident low‐traumatic (falls from a standing height or less) or non‐traumatic fractures, classified as MOFs (hip, clinical spine, forearm, or shoulder fractures) or other fractures, including peripheral ones, were registered during each annual phone call. All fractures whose origin was clearly traumatic were excluded (51 fractures, corresponding to 1.7% of all fractures). Pathological fractures were also excluded. All reported fractures were validated by written medical reports (radiographs and/or surgical reports) allowing to exclude “false positive” reports. Validated fractures not reported by study participants (“false negative” reports) were also included.^(^ ^15^, ^16^ ^)^

### Estimated fracture risk: FRAX and Garvan absolute fracture risk prediction

Baseline data were used to calculate the estimated 10‐year risk of fracture for each individual with the FRAX tool adapted for Belgium. To compare results at 5 years, we multiplied the 10‐year probabilities by 0.5 to convert to 5‐year probabilities. Leslie and colleagues^(^ ^17^ ^)^ showed indeed a near linear agreement between observed versus predicted (rescaled) FRAX probabilities at different follow‐up times.

We computed the 5‐year risk according to the Garvan model with BMD based on the FractureRiskCalculator.com website. In agreement with T Nguyen, in order to overcome some limitations when dealing with missing falls history data in the computation of Garvan score, we used the following falls classification: instead of 0, 1, 2 falls per year, we used 0, 1.5, and 4.2 for those with no falls, 1 to 3 falls, and 4 or more falls per year.

### National Osteoporosis Foundation (NOF) and Belgian thresholds for treatment strategies

The NOF threshold of 3% estimated 10‐year risk of hip fracture and 20% of major osteoporotic fracture, recommended for FRAX^(^ ^11^ ^)^ as cost‐effective for osteoporosis treatment, was applied to our cohort for both FRAX and Garvan tools.

We also applied the Belgian treatment strategy regarding the risk thresholds as follows: Treatment is indicated if the predicted 10‐year risk of hip fracture by FRAX or Garvan is ≥3% for individuals aged <70 years and ≥5% for individuals aged ≥70 years, or if the risk of MOF or “any Garvan fracture” is ≥20% independently of age.^(^ ^12^ ^)^

We calculated the sensitivity, specificity, the positive predictive value (PPV), negative predictive value (NPV), and number needed to treat (NNT) to prevent one fracture (hip, MOF, or “any Garvan” fracture).

PPV was calculated as the number of women who experienced fracture (hip, MOF or “any Garvan” fracture) and met treatment criteria divided by the total number of women who met treatment criteria. NPV was calculated as the number of women without fracture (hip, MOF, or “any Garvan” fracture) who did not meet treatment criteria divided by the total number of women who did not meet treatment criteria.

For NNT, we assumed that osteoporosis therapy would be undertaken by all women who qualified for treatment under this strategy/threshold and that pharmacotherapy would provide 30% relative risk reduction in fracture risk. NNT was calculated as 1 / (PPV × 0.3).

### Statistics

#### Calibration

The Hosmer‐Lemeshow test was used to estimate the quality of the calibration and calibrations plot results were drawn.^(^ ^18^ ^)^ A *p* value >0.05 for the Hosmer‐Lesmeshow test indicates that the data are well calibrated. The calibration plot, divided in deciles, was used to explore the quality of the predictions. Last, we performed linear regressions of the predicted risk on the observed risk percentages without forcing the intercept to go through the zero.

We investigated with an *F* test whether the observed slope of the linear regression was significantly different from 1 (the identity line), and the *p* value of the intercept indicates whether the intercept is different from zero. We compared the observed proportion of women who sustained a fracture with the proportion predicted by each calculator. These analyses were undertaken in the entire cohort and then repeated in the cohort divided into deciles of estimated fracture risk .

All analyses were performed using R version 3.6.2.^(^ ^19^ ^)^ All tests were two‐tailed, and *p* < 0.05 was considered significant.

#### ROC curve analysis

We used receiver operating characteristic (ROC) curve analysis to assess the ability of each calculator to discriminate between individuals who sustained hip, MOFs, or “any Garvan fractures”^(^ ^4^ ^)^ and those who did not. An area under the curve (AUC) of 0.50 indicates a result no better than chance, an AUC <0.6 poor discriminative value, 0.6 to 0.8 moderate discriminative value, and >0.8 high discriminative value.^(^ ^18^ ^)^ We compared the ROC curves between formulas using Delong's test^(^ ^20^ ^)^ to detect a difference in the quality of the prediction between two models. We used the software R, version 3.6.2,^(^ ^19^ ^)^ and its packages ROCR and pROC to perform the statistical analyses.

## Results

Of the 3560 participants enrolled in the study, data on 3030 women were available for analysis. A totoal of 530 women were censored because of loss of follow‐up (249 impossible to recontact, 221 deaths, 33 refusals to participate after the baseline visit, 27 moves abroad).

The baseline characteristics of our cohort are presented in Table 1. The clinical characteristics of censored subjects, such as body mass index, secondary osteoporosis, history of falls, and tobacco and alcohol consumption, did not differ from those included in the study (all p > 0.05). However, compared with the participating subjects, those lost at follow‐up were significantly older (*p* < 0.001) and tended to have more often a history of personal fractures (*p* = 0.02) or parental hip fracture (*p* = 0.03).

**Table 1:** Baseline Characteristics of the Population (*N* = 3030)

Variables	%	No. of subjects
Age ≥70 years	44.5	1347/3030
BMI < 20 kg/m^2^	8.0	243/3030
History of previous fracture	26.4	801/3030
Parental hip fracture	13.8	409/2955
Secondary osteoporosis (all causes; see below)	9.4	284/3030
Rheumatoid arthritis	1.0	31/3030
Current smoking	11.2	340/3030
Daily alcohol consumption ≥3 units	7.5	227/3030
Corticosteroids ≥3 months	7.5	226/3030
Causes of secondary osteoporosis		
Early non‐substituted menopause	5.4	165/3030
Untreated hyperthyroidism	1.6	48/3030
Early menopause induced by gynecologic cancer	0.5	14/3030
Inflammatory bowel diseases	0.5	14/3030
Type 1 diabetes	0.3	8/3030
Chronic malnutrition, anorexia	0.2	6/3030
Osteogenesis imperfecta, chronic liver disease	0	0/3030
Breast cancer + aromatase inhibitor therapy	1.2	35/3030
Chronic obstructive pulmonary disorder	1.4	41/3030
Prolonged immobilization	0.8	25/3030
Hyperparathyroidism	0.2	7/3030
Other endocrine causes	0.1	3/3030
Organ transplantation	0.1	3/3030
Use of PPIs	23.6	411/1739
Use of SSRIs	29.2	163/559
Hypnotics intake	31.0	935/3016
Sedentary lifestyle	5.0	153/3030
History of fall(s)	19.5	592/3030
Time to last fall		
Fall in the previous month	5.1	156/3030
Fall between 1 and 6 months	15.0	453/3030
Frequency of falls		
Less than one per month	17.6	533/3030
More than one per month but less than one per week	1.9	58/3030
More than one per week	0.03	1/3030

BMI = body mass index; PPIs = proton pump inhibitors; SSRIs = selective serotonin reuptake inhibitors.

During a 5‐year follow‐up period, 9% to 12% of participants experienced an incident fracture, and 1.6% experienced an incident hip fracture (Table 2). The estimated fracture risks during follow‐up for hip and other osteoporotic fractures for each calculator in the entire cohort are shown in Table 2.

**Table 2:** Proportion of Women With Incident Fractures in the FRISBEE Cohort and Estimated Fracture Risk by Garvan and FRAX in the Entire Cohort

Fracture type	Incident fracture FRISBEE (*n*, %)	Predicted fractures at 5 years in the entire cohort (*N* = 3030)	
		Garvan (*n*, %)	FRAX (*n*, %)
Hip	47 (1.5)	93 (3.1)	46 (1.5)
FRAX‐MOFs	281 (9.3)	N/A	160 (5.3)
Any Garvan fractures	356 (11.7)	306 (10.1)	NA

MOF = major osteoporotic fracture; N/A = not applicable.

FRAX‐MOFs = hip, wrist, proximal humerus, and clinical spine; any Garvan fractures = hip, vertebra (symptomatic), wrist, metacarpal, humerus, scapula, clavicle, distal femur, tibia/fibula, patella, pelvis, ribs, sternum, and hands and feet, excluding digits.

### Calibration

**Fig 1:**
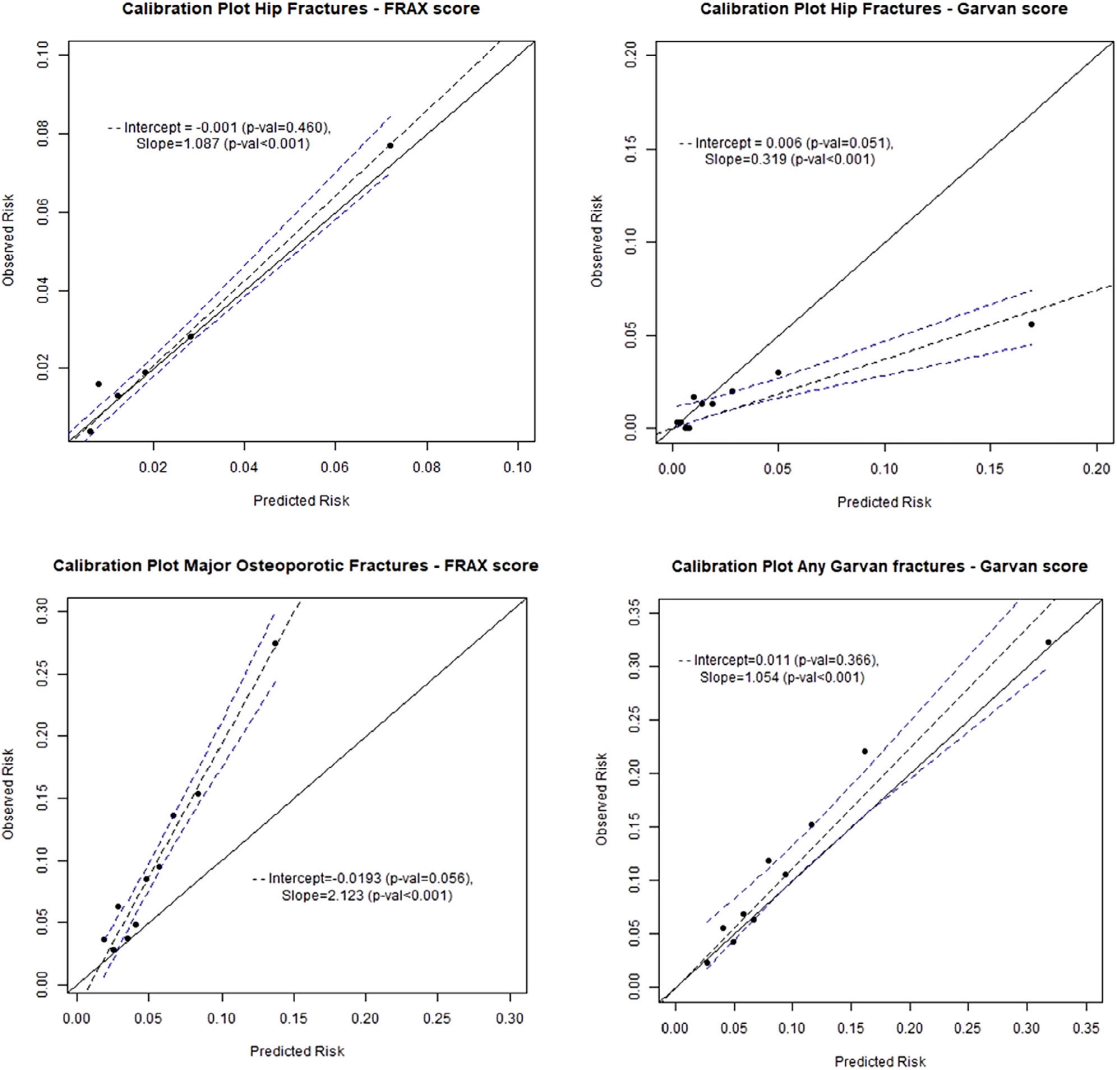
Calibration of the calculators. Each panel shows a plot of the observed probability of fracture versus the mean estimated fracture probability for the cohort divided by decile of estimated probability. The solid line represents a perfectly calibrated model, and the dotted lines the line of best fit with its 95% confidence interval.

Calibration plots were generated for calculator‐defined hip fracture for MOFs and for any Garvan fractures (Fig. 1).

For hip fractures (FRAX), the slope of the regression coefficient was 1.09 (*p* < 0.001; intercept −0.001, *p* = 0.46), showing that the FRAX tool for hip fracture was well calibrated, with overall risk estimates close to observed fracture rates (Fig. 1). The Garvan tool largely overestimated hip fracture incidence, especially for elevated estimated risks of fracture. The calibration slope was only 0.32 (*p* < 0.001; intercept 0.006, *p* = 0.05). For “any Garvan” fractures, the Garvan calculator appeared to be well calibrated across deciles of estimated fracture risk, with a slight underestimation of fracture events (slope 1.05, *p* < 0.001; intercept 0.01, *p* = 0.37), whereas FRAX tended to consistently underestimate the observed incidence of MOFs (slope 2.12, *p* < 0.001; intercept −0.02, *p* = 0.06) (Fig. 1).

### Discrimination/ ROC analyses

**Fig 2:**
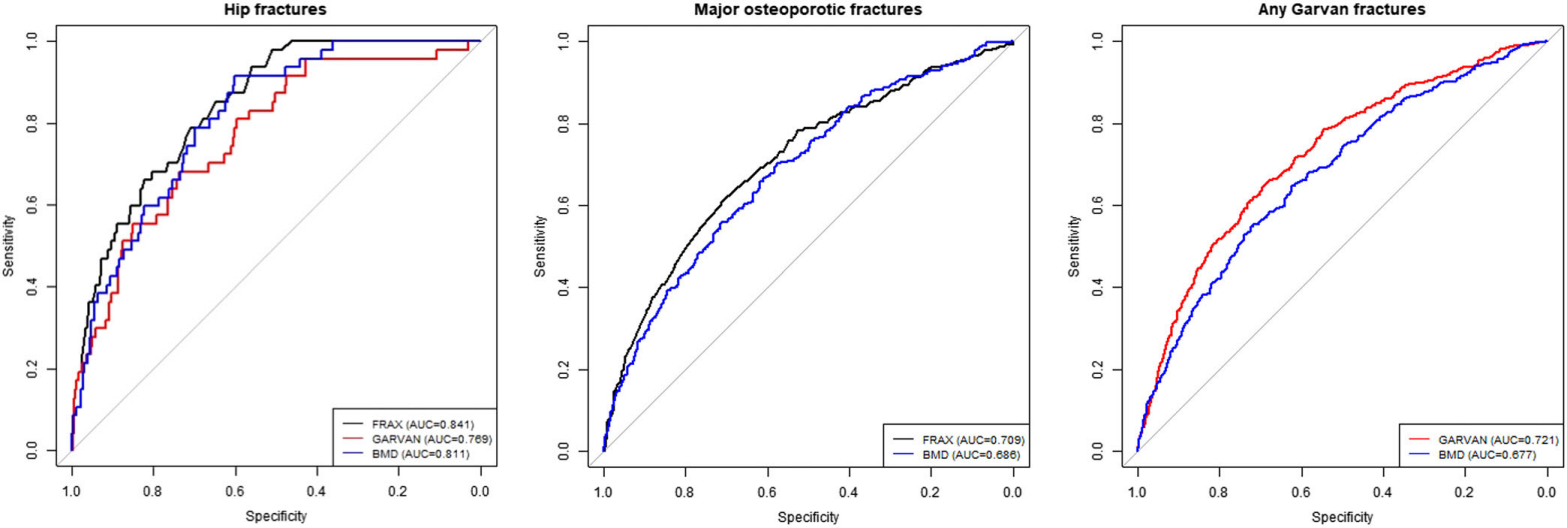
Receiving operating characteristics (ROC) curves for hip and calculator‐defined osteoporotic fractures.

ROC curve analysis was performed to assess the discrimination power for prediction of hip fracture alone and MOFs or “any Garvan” fractures for both calculators (Fig. 2). FRAX had the highest AUC for hip fracture prediction (0.841, 95% confidence interval [CI] 0.795–0.887), followed by BMD (0.811, 95% CI 0.761–0.862). Garvan AUC for hip fracture prediction (0.769, 95% CI 0.702–0.836) was lower. The Delong's test for two correlated curves showed that FRAX had a significantly higher AUC than Garvan (*p* = 0.01), but it was not significantly different than the BMD AUC (Table 3).

**Table 3:** Area Under the Curve (AUC) for Receiver Operating Characteristic Curves for Each Calculator and BMD

Calculator/variable	Hip fractures AUC (95% CI)	MOFs according to FRAX AUC (95% CI)	Garvan‐defined osteoporotic fractures AUC (95% CI)
FRAX	0.841^a^ (0.795–0.887)	0.708 (0.675–0.741)	N/A
GARVAN	0.769 (0.702–0.836)	N/A	0.721^b^ (0.693–0.749)
BMD	0.811 (0.761–0.862)	0.686 (0.653–0.718)	0.678 (0.647–0.707)

BMD = bone mineral density; CI = confidence interval; MOF = major osteoporotic fracture; N/A = not applicable.

Data are area under the curve (95% confidence interval).

^a^ *p* value 0.01 (FRAX's AUC compared with GARVAN's AUC).

^b^ *p* value <0.001 (Garvan's AUC compared with BMD's AUC).

The discriminatory power for prediction of MOFs or “any Garvan” fractures was lower than those for hip fracture prediction for both models. Garvan AUC for “any Garvan” fractures prediction was the highest (0.721, 95% CI 0.693–0.749), significantly higher than BMD's AUC (0.678, 95% CI 0.647–0.707; *p* < 0.001). The difference was not significant when comparing BMD/AUC for MOFs and FRAX prediction for MOFs (0.708, 95% CI 0.675–0.741, *p* = 0.08) (Table 3).

### NOF and Belgian thresholds for treatment selection

In the whole cohort, the prevalence of hip fracture was 1.5%, that of a MOF 9.3%, and that of “any Garvan” fractures 11.7%. Without any selection criteria, the number of patients needed to treat to avoid one hip fracture would be 215, under the hypothesis that a treatment would prevent 30% of the fractures.

By applying the NOF hip fracture probability thresholds (3% independently of age) for selecting candidates for treatment, FRAX selected for treatment 29% (*n* = 870/3030) of the subjects and Garvan 42% (*n* = 1270/3030). The NNT to prevent one hip fracture with NOF threshold of 3% for all ages would be 81 for FRAX and 111 for Garvan. The false negative rate was at 23% (*n* = 11/47) for FRAX and 19% (*n* = 9/47) for Garvan (Table 4).

**Table 4:** Prediction of Incident Hip Fracture, Incident Major Osteoporotic Fracture, and Any Garvan Fracture, Under National Osteoporosis Foundation (NOF) and Belgian Strategy

	True positive	True negative	False positive	False negative	Sensitivity	Specificity	PPV	NPV	NNT	Avoided fractures meeting treatment criteria
FRAX hip (Belgian guidelines)	33	2411	572	14	70%	81%	5%	99%	61	10
FRAX hip (NOF guidelines)	36	2149	834	11	77%	72%	4%	99%	81	11
Garvan hip (Belgian guidelines)	34	1968	1015	13	72%	66%	3%	99%	103	10
Garvan hip (NOF guidelines)	38	1751	1232	9	81%	59%	3%	99%	111	11
FRAX MOF	75	2553	196	206	26%	93%	28%	99%	12	23
Any Garvan fractures	97	2476	198	259	27%	93%	34%	91%	10	29

PPV = positive predictive value; NPV = negative predictive value; NNT = number needed to treat under the hypothesis that the treatment would prevent 30% of the fractures; MOF = major osteoporotic fracture.

Belgian guidelines: treatment is indicated if the predicted 10‐year risk of hip fracture by FRAX or Garvan is ≥3% for individuals aged <70 years and ≥5% for individuals aged ≥70 years, or if the risk of MOF or “any Garvan fracture” is ≥20% independently of age. National Osteoporosis Foundation (NOF) strategy: treatment is indicated if the predicted 10‐year risk of hip fracture by FRAX or Garvan is ≥3% for individuals, or if the risk of MOF or “any Garvan fracture” is ≥20% independently of age.

Applying a mixed threshold (as proposed by the NOGG), which was adapted by the Belgian Bone Club (a hip fracture probability of 3% before 70% or 5% if older), 20% (*n* = 605/3030) of the participants would have been selected for treatment by the FRAX and 35% (*n* = 1049/3030) by Garvan. Among these women, only 5.4% (*n* = 33/605) identified by FRAX score and 3.2% (*n* = 34/1049) by Garvan sustained a hip fracture.

The NNT to prevent one hip fracture under the Belgian strategy would be 61 for FRAX and 103 for Garvan. Thirteen (Garvan) (28%) or 14 (FRAX) subjects (30%) who eventually underwent a fracture would not have been treated (false negatives).

For MOFs, FRAX selected 8.9% (*n* = 271/3030) of candidates for treatment with only 27.3% (*n* = 75/275) of true positive fractures. For “any Garvan” fracture, Garvan score selected 9.7% (*n* = 295/3030) candidates for treatment with a true positive rate of 32.9% (*n* = 97/295) (Table 4). The NNT would thus have been 12 using FRAX and 10 using Garvan. Two hundred six (FRAX) (73.3%) or 259 (Garvan) patients (72.8%) who eventually suffered a fracture would not have been treated.

## Discussion

Most guidelines on postmenopausal osteoporosis recommend the estimation of the individualized absolute fracture risk and treatment of high fracture risk individuals only.^(^ ^12^ ^)^ It is thus of utmost importance to determine if available prognostic tools accurately predict risk of fracture in independent prospectively studied populations. Underestimating fracture risk will lead indeed to false reassurance and treatment abstention in patients in whom fractures could have been avoided. On the other hand, overestimation of fracture risk may lead to overtreatment in the population, increasing the cost/benefit ratio.

In this study, we evaluated the calibration and predictive accuracy of the FRAX and Garvan tools in a prospective cohort of volunteer, postmenopausal women followed for at least 5 years.

The Belgian FRAX tool appeared to be well calibrated for hip fracture but not for MOFs, where it consistently underestimated the incidence of fracture. Other studies already reported that FRAX underestimates osteoporotic fractures risk.^(^ ^21^, ^22^ ^)^ However, a validation study conducted in Canada by Leslie and colleagues^(^ ^23^ ^)^ showed Canadian FRAX to be well calibrated for both hip fractures and MOFs. These differences in calibration for MOFs probably result from the fact that the Canadian FRAX was calibrated using MOF/hip ratios from the US FRAX tool (version 3.0)^(^ ^24^, ^25^ ^)^ and not based on Swedish ratios. Calibration of the Canadian FRAX tool with Swedish ratios would have underestimated predicted MOF risk^(^ ^23^ ^)^ since Swedish MOF/hip fracture ratios were 23% lower compared with those used in Canadian FRAX calibration.^(^ ^25^ ^)^ The Belgian FRAX was calibrated using Swedish reference standards,^(^ ^10^ ^)^ and the Swedish MOF/hip ratio also appears to be substantially lower than in our cohort.^(^ ^26^ ^)^ In another study, Leslie and colleagues evaluated the performance of eight national FRAX tools for fracture prediction in Canadian women, showing good calibration for hip fracture prediction for Canadian and most country‐specific FRAX tools, with the exception of Sweden (overestimation of fracture risk) and China (underestimation of fracture risk). Regarding MOF prediction, greater between‐country variations were observed, with Sweden FRAX and China FRAX having the largest over‐ and underestimation, respectively, in the Canadian cohort.^(^ ^27^ ^)^

The Garvan tool was well calibrated for “any Garvan” fractures in our cohort but largely overestimated the hip fractures, in agreement with the findings of Bolland and colleagues.^(^ ^21^ ^)^

As illustrated by the areas under receiver operating characteristics curves (Fig. 2), both models tended to predict better hip fractures than MOFs or “any Garvan” fractures.

These findings for the FRAX calculator are similar to those reported by Leslie and colleagues, who evaluated the FRAX‐Canada in an independent cohort and found an AUC of 0.83 for hip fractures and AUC of 0.69 for MOFs.^(^ ^23^ ^)^ Similar results were also reported in other studies: Bolland and colleagues found an AUC of 0.70 for the hip and 0.64 for MOFs in New Zealand^(^ ^21^ ^)^; in the nine development cohorts, FRAX with BMD had AUC values of 0.78 for hip fracture and 0.63 for MOFs, and in the 11 validation cohorts, FRAX with BMD had a median (range) AUC of 0.74 (0.65–0.81) for hip fractures and 0.60 (0.55–0.77) for MOFs.^(^ ^5^ ^)^ The better performance of both models to predict hip fractures than other fractures could be due in part to the fact that in both models BMD is measured at the femoral neck and the gradient risk (GR) of hip fractures with decreasing femoral BMD is higher than for any other fracture.

Kanis and colleagues showed that BMD alone predicted hip fracture with a gradient risk of 2.6/SD and other osteoporotic fractures with a GR of 1.6/SD.^(^ ^5^ ^)^

The FRAX tool was more discriminant than Garvan with regard to hip fractures. The predictive value of both models was not significantly superior to BMD alone. Only for “any Garvan” fractures the Garvan AUC was significantly superior when compared with BMD AUC alone.

This lower discriminatory performance for hip fracture of the Garvan calculator in our cohort is similar to that found by Bolland and colleagues (AUC of 0.69 for hip and 0.64 for “any Garvan” fractures).^(^ ^21^ ^)^

Correction for the calibration factor did not change the discriminatory power of the two models in terms of AUC but would change the optimal thresholds.

Using the NOF and proposed Belgian intervention thresholds for both calculators in our cohort showed a relatively low sensitivity and specificity for predicting incident hip, MOF, or any Garvan fracture, suggesting a low degree of accuracy in the identification of women who will sustain a fracture. The percentage of subjects who suffered a hip fracture and would not have been selected for treatment (false negative) was slightly higher for the age‐specific treatment threshold proposed by the Belgian algorithm compared with the fixed threshold recommended by NOF guidelines but with a lower number needed to treat.

There are several limitations to our study. The cohort was a group of older women and the results may not be applicable to men or to younger women. The results are based on a 5‐year follow‐up, assuming linearity of FRAX with follow‐up time. This is a reasonable assumption according to Leslie and colleagues,^(^ ^17^ ^)^ but which shall be verified only when our 10‐year follow‐up data will be available. Approximately 20% of women used bisphosphonates at some point during follow‐up. Such therapy may have prevented fractures in these participants and potentially caused the calculators to overestimate the fracture risk. Significant differences in the predicted risk of fracture between the prediction tools could also be due to variations in the input or output variables and model construction.^(^ ^28^ ^)^ The FRAX tool calculates a probability of fracture, taking into account the competing risk of mortality, whereas the Garvan calculator gives a cumulative incidence, without taking into account mortality risk. Also, the observed probability might be influenced by the recency of prior fractures, which is not currently captured by the available fracture risk calculators.^(^ ^29^ ^)^

A strength of our study is that results were derived from an independent representative population not involved in the development of Garvan or FRAX tools. Our data provide useful information on the accuracy of these prognostic models in clinical practice and also emphasize the importance of carefully comparing the performance of prediction tools in the same population. The ascertainment of fractures was systematic, and all osteoporotic fractures were validated by a radiology and/or a surgical report in order to minimize any misclassification.^(^ ^15^, ^16^ ^)^

In conclusion, our data indicate that, in terms of calibration, the Belgian FRAX estimated quite well hip fractures but not major osteoporotic fractures, while the Garvan calculator appeared to better estimate “any Garvan” fractures. Both models had a good discriminatory value for hip fractures but only a moderate discriminatory ability for MOFs or “any Garvan” fractures.

Regarding the predictive accuracy, all current models for fracture risk assessment are suboptimal and the challenge is to find ways to improve this accuracy in part by incorporating new markers for fracture risk (such as quality of bone, falls) and by adopting new modeling strategies.

## Disclosures

We declare that there are no known conflicts of interest associated with this publication that could be perceived as prejudicing the impartiality of the research reported. MRL reports consultancy and lecture fees from Alexion, Amgen, Kyowa Kirin, Menarini, Sandoz, Takeda, UCB, and Will Pharma, unrelated to this work. JJB reports consulting fees from Sandoz, Takeda, and UCB, unrelated to this work.

## Authors' roles

FB, PB and JJB contributed to the study design. AC, VK collected data. AC, JFF, MM and MP analysed data. FB wrote the first draft ofthe manuscript. LI, MS, BF (Benoit F), AM, RK, MRL, PB and JJB critically revised the manuscript. FB, LI, AC, VK, JFF, MM, RK, MS, BF, AM, MP, MRL, PB, JJB all approved the draft of the manuscript.

### Peer Review

The peer review history for this article is available at https://publons.com/publon/10.1002/jbm4.10532.
